# Supplementary material for: Hysteresis in the Thermo-Responsive Assembly of Hexa(ethylene glycol) Derivative-Modified Gold Nanodiscs as an Effect of Shape
Source: Nanomaterials (Basel). 2022 Apr 21;12(9):1421. doi: 10.3390/nano12091421 (PMC9102705; doi:10.3390/nano12091421)
Supplement: Supplementary file 1 [file nanomaterials-12-01421-s001.zip › nanomaterials-1681216-supplementary.pdf]

nanomaterials

Supplementary Materials

Hysteresis in the thermo-responsive assembly of hexa(ethylene glycol) derivative-modified gold nanodiscs as an effect of shape

Joshua Chidiebere Mba,[1] Hideyuki Mitomo,[2,*] Yusuke Yonamine,[2] Guoqing Wang,[3] Yasutaka Matsuo,[2] and Kuniharu Ijiro,[2,*]

[1] Graduate School of Life Science, Hokkaido University, Kita 10, Nishi 8, Kita-Ku, Sapporo, Hokkaido 060-0810, Japan

[2] Research Institute for Electronic Science, Hokkaido University, Kita 21, Nishi 10, Kita-Ku, Sapporo, Hokkaido 001-0021, Japan

[3] College of Food Science and Engineering, Ocean University of China, 5 Yushan Road, Qingdao 266003, China

* Correspondence: e-mail: mitomo@es.hokudai.ac.jp (H.M.), ijiro@es.hokudai.ac.jp (K.I.)

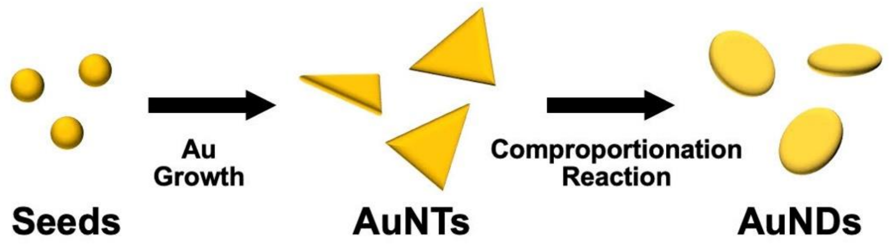

Scheme 1. Illustration of AuND synthesis.

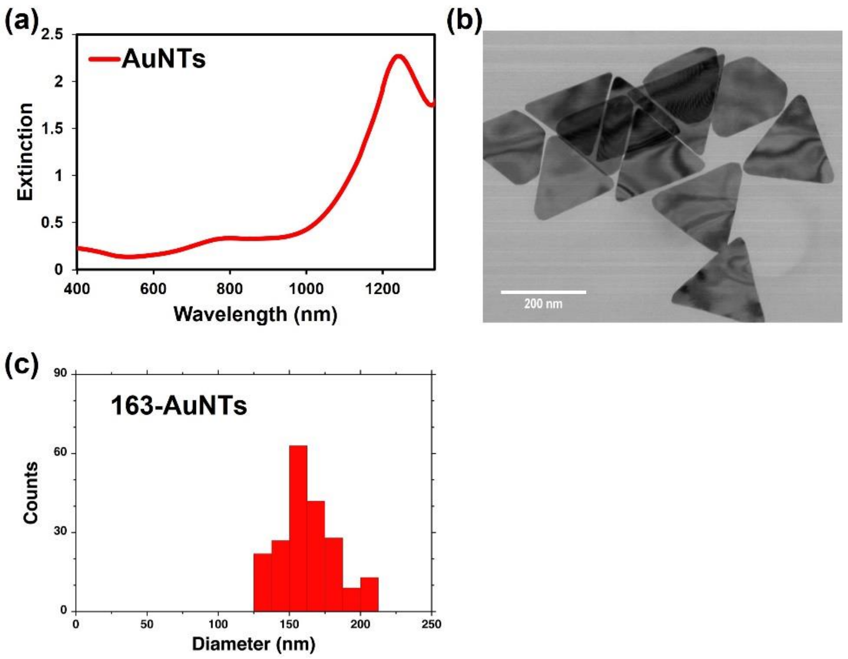

Figure S1. Characterization of gold nanotriangles (AuNTs). (**a**) Extinction spectrum of AuNTs. (**b**) STEM image of AuNTs. (**c**) Histogram of AuNT edge length.

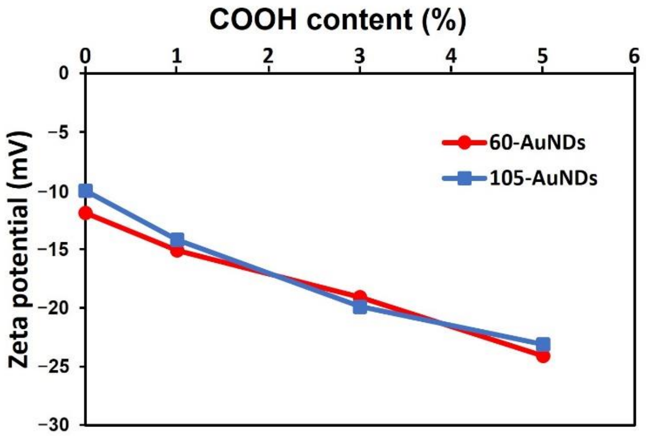

Figure S2. A plot of the zeta potential of AuNDs against the **COOH** content on their surface ligands.

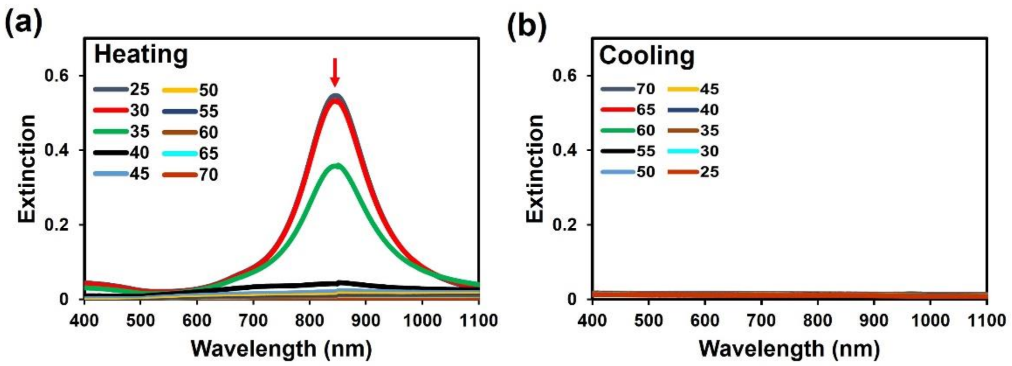

Figure S3. Thermo-responsive assembly of 105-AuND-(100:0). (**a**) The extinction spectra of 105-AuND-(100:0) upon heating from 25 to 70 °C and (**b**) upon cooling from 70 to 25 °C.

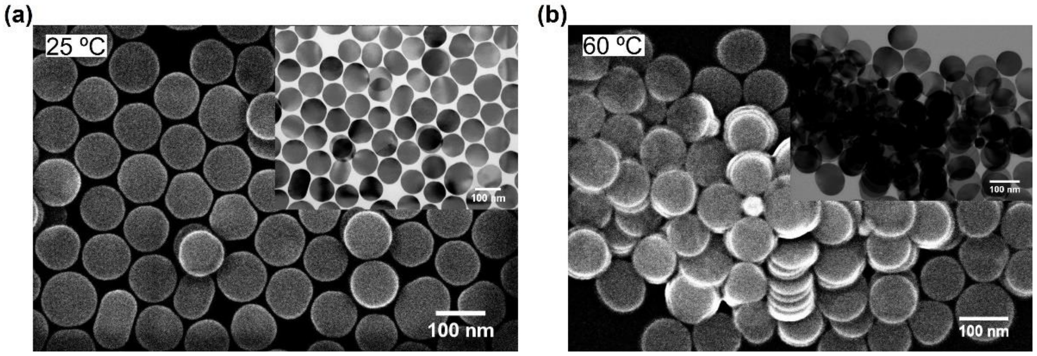

Figure S4. STEM images of 105-AuND-(97:3). (**a**) SEM image of 105-AuND-(97:3) dried at 25 °C and (**b**) dried at 60 °C with a STEM image as the insert.

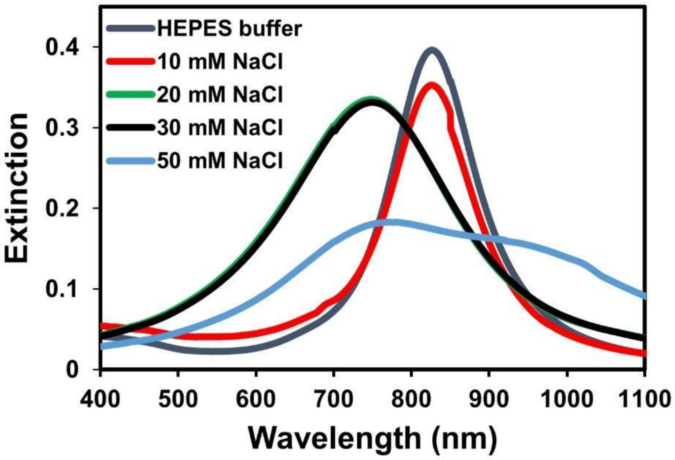

Figure S5. The extinction spectra of 105-AuND-(97:3) under various salt concentrations.

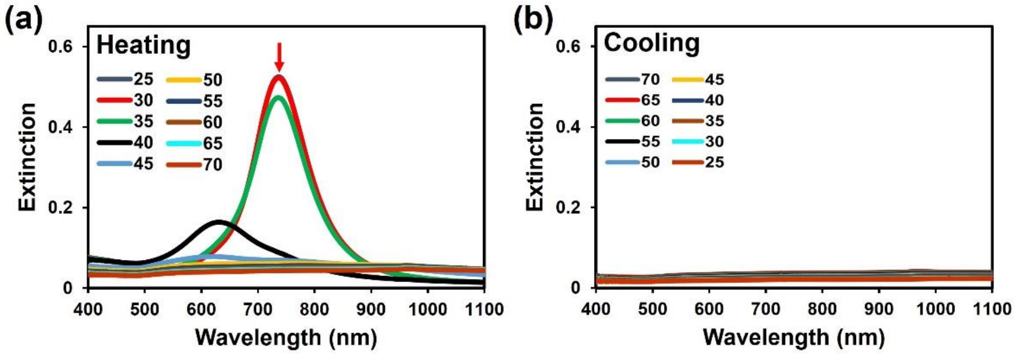

Figure S6. Thermo-responsive assembly of 60-AuND-(100:0). (**a**) The extinction spectra of 60-AuND-(100:0) upon heating from 25 to 70 °C and (**b**) upon cooling from 70 to 25 °C.

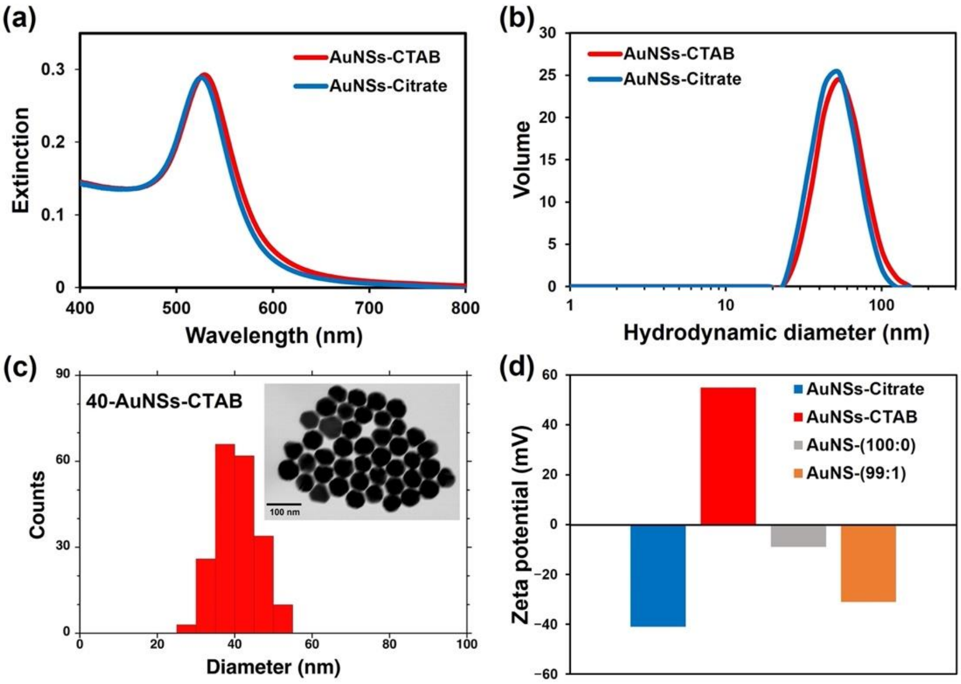

Figure S7. Characterization of 40-AuNSs. (**a**) The extinction spectra of 40 nm citrate-stabilized AuNSs before (red) and after (blue) CTAB coating. (**b**) DLS analyses of 40 nm citrate-stabilized

AuNSs before (red) and after (blue) CTAB coating. (**c**) TEM image and size distribution of 40 nm CTAB-capped AuNSs. (**d**) Zeta potentials of 40-AuNSs stabilized with citrate (blue), CTAB (red) and modified with a mixture of **C1:COOH** ligands; (100:00) (grey) and (99:1) (orange).

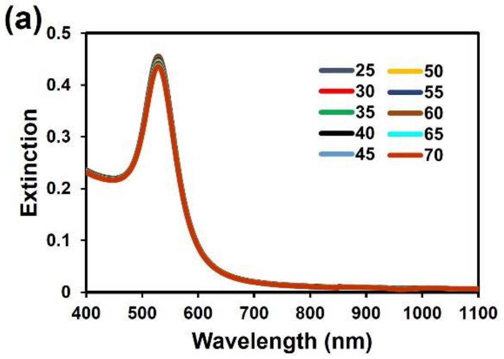

Figure S8. Thermo-responsive assembly of 40-AuNS-(99:1). (**a**) The extinction spectra of 40-AuNS-(99:1) upon heating from 25 to 70 °C and (**b**) upon cooling from 70 to 25 °C.

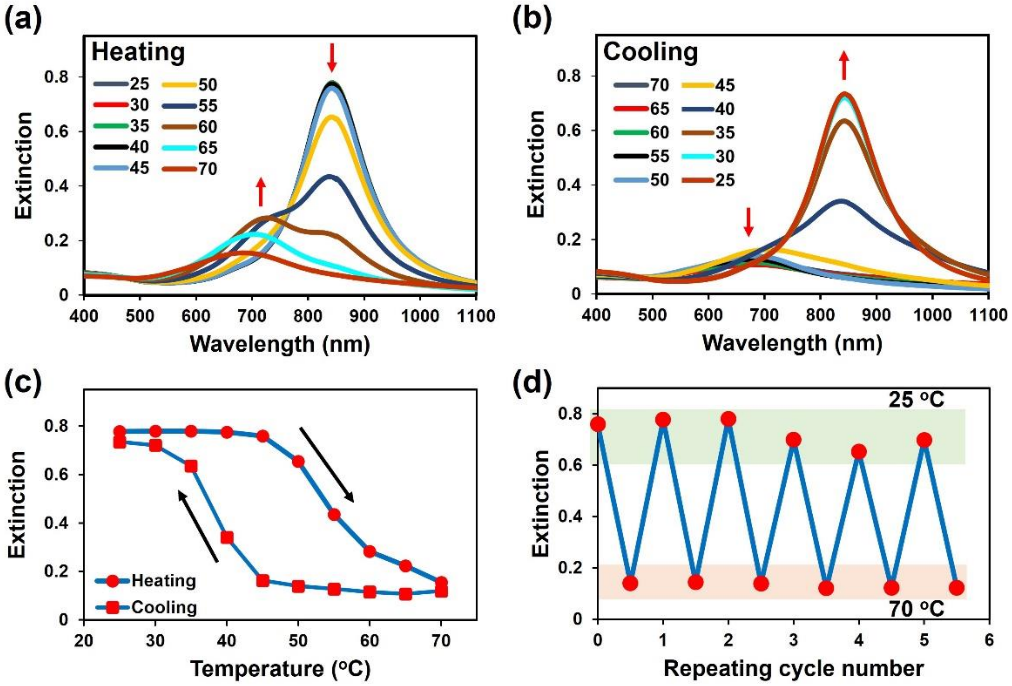

Figure S9. Thermo-responsive assembly of 105-AuND-(99:1). (**a**) The extinction spectra of 105-AuND-(99:1) upon heating from 25 to 70 °C and (**b**) upon cooling from 70 to 25 °C. (**c**) The extinction values at 827 nm against temperature changes. (**d**) The reversible changes in extinction values between 25 and 70 °C.

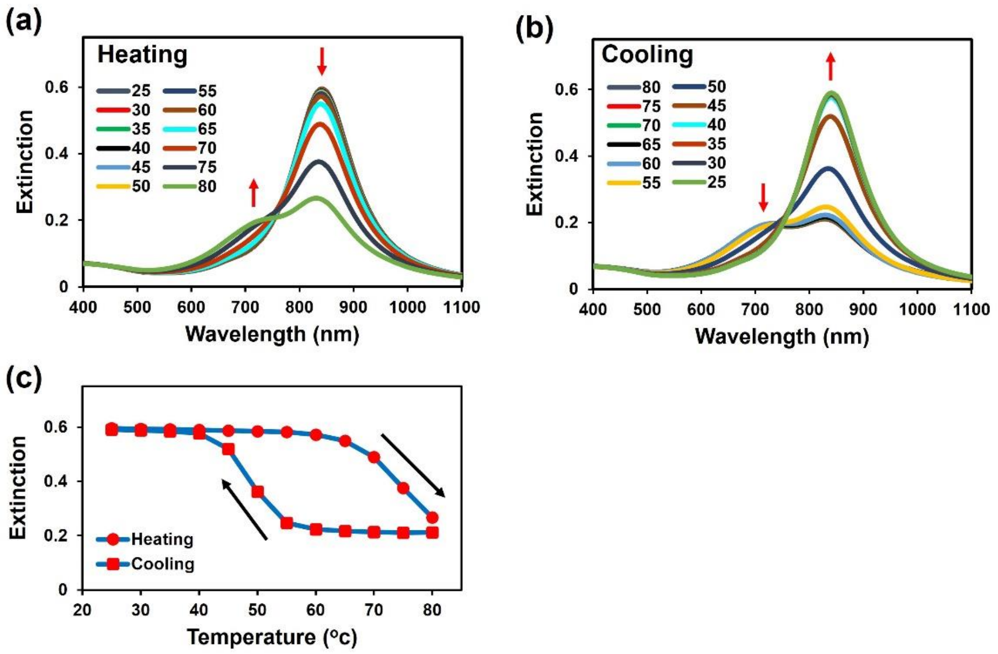

Figure S10. Thermo-responsive assembly of 105-AuND-(95:5). (**a**) The extinction spectra of 105-AuND-(95:5) upon heating from 25 to 80 °C and (**b**) upon cooling from 80 to 25 °C. (**c**) The extinction values at 827 nm against temperature changes.

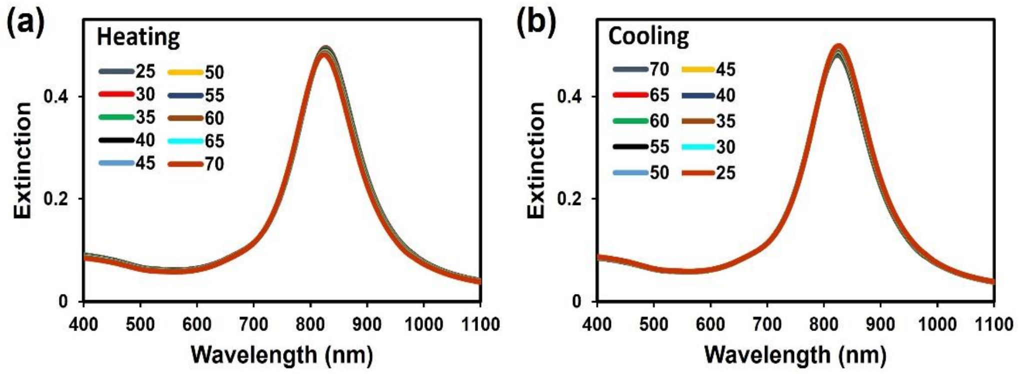

Figure S11. Thermo-responsive assembly of 105-AuND-(90:10). (**a**) The extinction spectra of 105-AuND-(90:10) upon heating from 25 to 70 °C and (**b**) upon cooling from 70 to 25 °C.

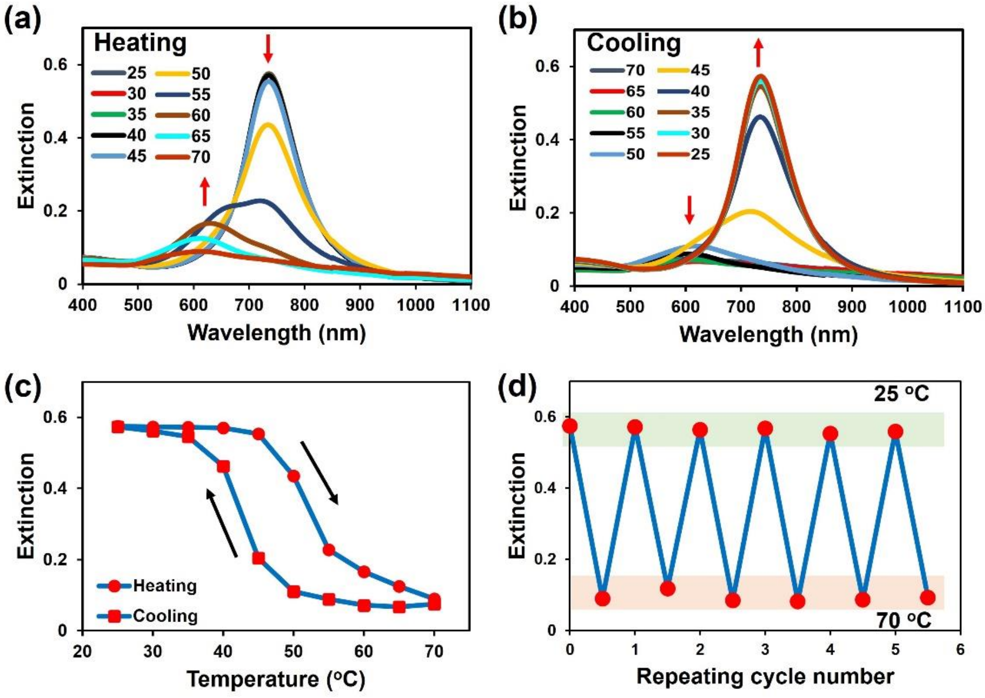

Figure S12. Thermo-responsive assembly of 60-AuND-(99:1). (**a**) The extinction spectra of 60-AuND-(99:1) upon heating from 25 to 70 °C and (**b**) upon cooling from 70 to 25 °C. (**c**) The extinction values at 715 nm against temperature changes. (**d**) The reversible changes in extinction values between 25 and 70 °C.

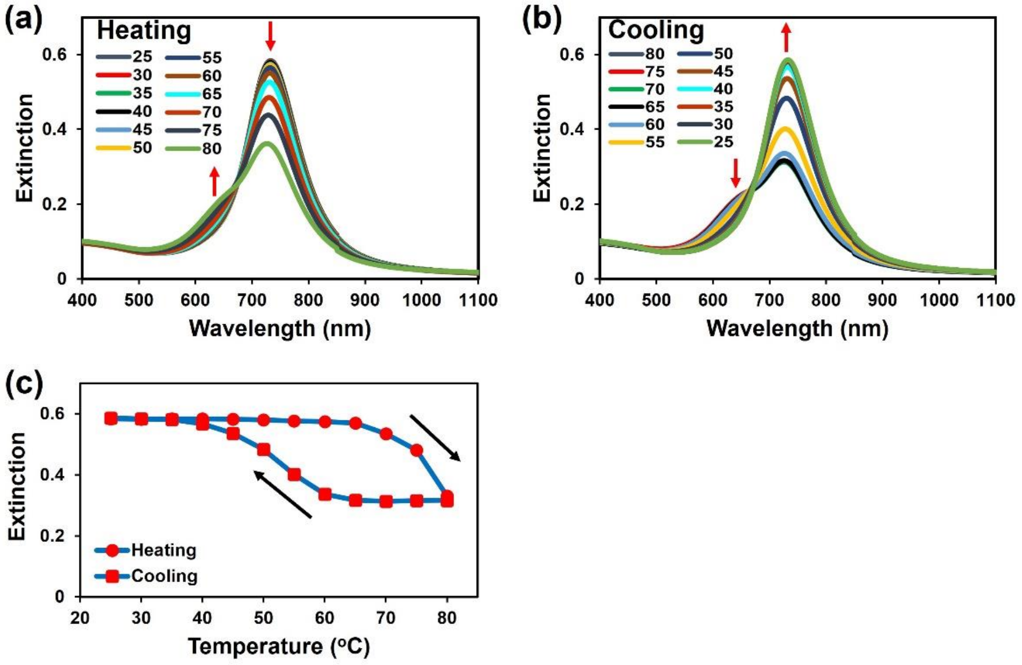

Figure S13. Thermo-responsive assembly of 60-AuND-(95:5). (**a**) The extinction spectra of 60-AuND-(95:5) upon heating from 25 to 80 °C and (**b**) upon cooling from 80 to 25 °C. (**c**) The extinction values at 715 nm against temperature changes.

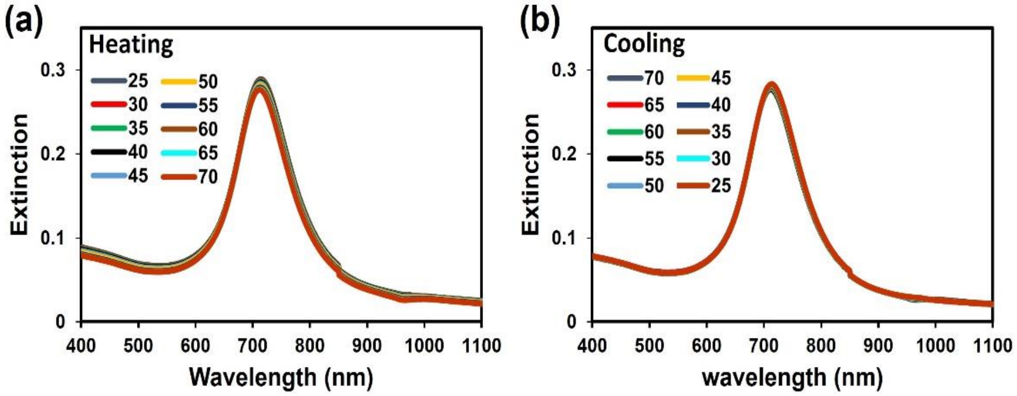

Figure S14. Thermo-responsive assembly of 60-AuND-(90:10). (**a**) The extinction spectra of 60-AuND-(90:10) upon heating from 25 to 70 °C and (**b**) upon cooling from 70 to 25 °C.

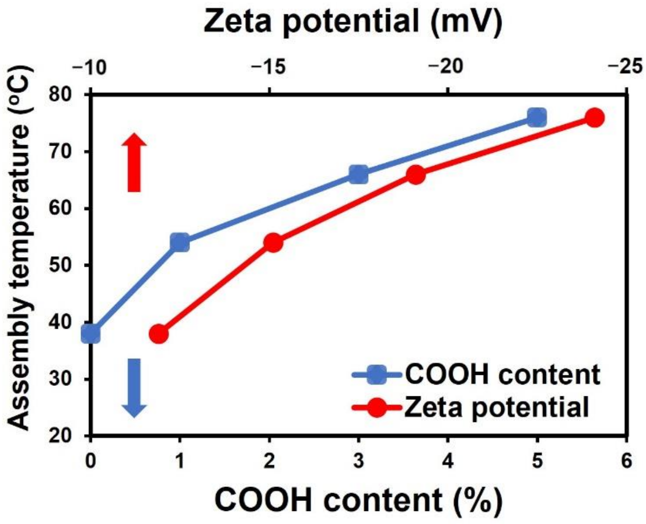

Figure S15. Assembly temperature of 60-AuNDs against **COOH** content and zeta potential.

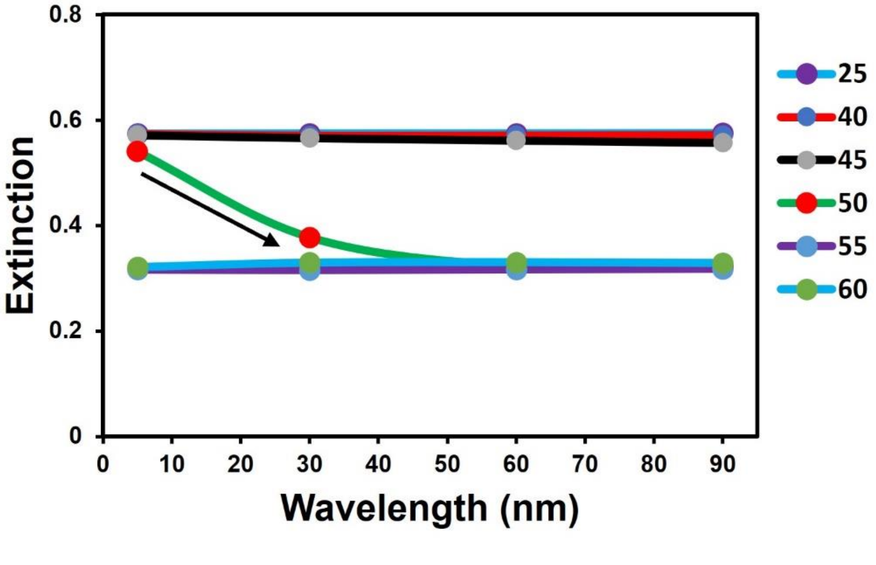

Figure S16. The plot of changes in extinction during the time course experiment of 60-AuND-(97:3) at 25, 40, 45, 50, 55 and 60 °C. The starting point was 25 °C and the sample was heated up to 90 min.

This was followed by heating at 40, 45, 50, 55 and 60 °C up to 90 min while measuring the spectrum change every 30 min.

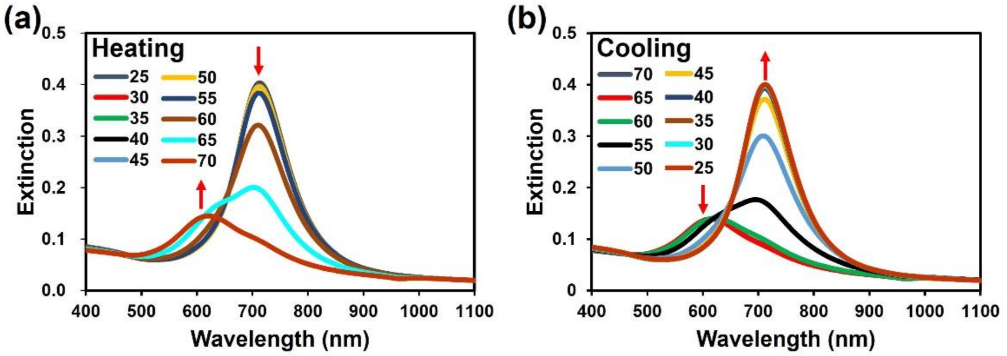

Figure S17. Thermo-responsive assembly of 60-AuND-(97:3) with 30 min of waiting time. (**a**) The extinction spectra of 60-AuND-(97:3) upon heating from 25 to 70 °C and (**b**) upon cooling from 70 to 25 °C.

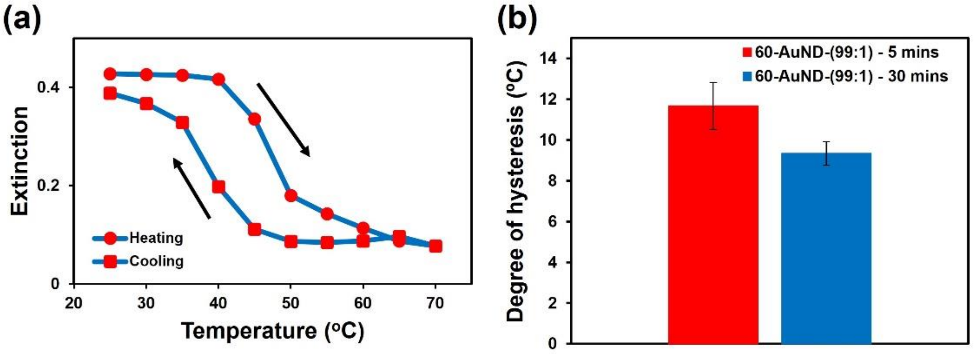

Figure S18. (**a**) The extinction values at 715 nm against temperature changes with 30 min waiting time for 60-AuND-(99:1). (**b**) A plot of the degree of hysteresis with the 5 and 30 min waiting time for 60-AuND-(99:1). The error bars represent standard deviation of the mean (n = 3).
